# Digital solutions to promote adolescent mental health: Opportunities and challenges for research and practice

**DOI:** 10.1371/journal.pmed.1004008

**Published:** 2022-05-31

**Authors:** Jason Bantjes

**Affiliations:** 1 Alcohol, Tobacco and Other Drug Research Unit, South African Medical Research Council, Cape Town, South Africa; 2 Institute for Life Course Health Research, Department of Global Health, Stellenbosch University, South Africa

## Abstract

Jason Bantjes discusses the accompanying study by Michelle Torok, Jin Han, and colleagues investigating the effects of a self-guided smartphone application on suicidal ideation among young adults.

The Coronavirus Disease 2019 (COVID-19) has had far-reaching consequences, precipitating dramatic increases in the global prevalence of depression and anxiety [1] while also spurring rapid advances in the use of digital technologies to deliver psychological interventions [2]. The need for social distancing and the increased prevalence of psychological distress that came with the pandemic have driven up the demand for digital solutions to mental health problems. Indeed, digital technologies have considerable potential to increase access to treatment and expand the range of available interventions. Digital solutions may also be particularly appropriate for expanding adolescent mental health services. Smartphone applications (apps) are one example of how technology can be used to promote adolescent wellness, as aptly illustrated in the accompanying research study in *PLOS Medicine* by Torok and colleagues, which investigated whether the LifeBuoy app could help reduce the severity of suicidal ideation among young adults [3]. This study adds to the small but growing body of research demonstrating the potential for novel technologies to transform adolescent mental health service delivery.

Apps are uniquely positioned to overcome some of the challenges typically associated with providing adolescent mental health services, including scheduling problems (i.e., finding convenient times between academic and social commitments to access services that typically only operate during business hours), adolescents’ reluctance to seek help from professionals, and their developmentally appropriate need for autonomy and independence [4]. Apps allow greater anonymity, thus helping to overcome stigma, while also providing greater control and choice over when and how users access psychological support [5]. There are potential economic advantages to using mental health apps [6], suggesting that they could help scale up adolescent services, especially in resource-constrained environments [7].

There is increasing evidence to support adolescents’ use of apps for a range of problems, including depression and anxiety [8], and apps could be a novel way to mitigate the increasing burden of adolescent suicidal behaviors. Torok and colleagues have now shown that apps may be an effective strategy to address suicide risk in young people [3]. In their randomized controlled trial, Torok and colleagues showed that 6 weeks of engagement with LifeBuoy, a self-guided app based on the principles of dialectical behavior therapy (DBT), led to reduced suicidal ideation among young adults (18 to 25 years old) compared to a visually similar but nontherapeutic control app [3]. DBT is an evidence-based psychotherapy focused on developing skills such as mindfulness, distress tolerance, emotional regulation, and maintaining interpersonal relationships. While this study contributes to the body of promising emerging evidence on the use of mental health apps, there is currently insufficient evidence to support the use of apps as a stand-alone self-management tool to promote adolescent mental health [9].

There are several challenges to realizing the full potential of app-based adolescent mental health interventions, including the ever-expanding digital divide and the need to ensure the benefits of digital interventions are shared with adolescents in low- and middle-income countries [9]; the need to involve adolescents in the design of apps to improve usability and acceptability [10]; the need for guidelines and regulations on consent, minimum safety standards, and data protection as well as ensuring that data privacy agreements are written in clear language that adolescents understand [11]; and the need for well-controlled studies of both the benefits and harms of apps by researchers who have no commercial interest in the products. Ethically, it might be argued that adolescents deserve special protection and that guidelines or regulations should be more stringent when apps are designed for or marketed and prescribed to adolescents. One of the notable aspects of the Torok and colleagues study is that the LifeBuoy app was designed with feedback from young people with lived experience of suicidal behavior, a factor that the authors note may have contributed to the high levels of engagement and favorable outcomes observed [3].

It is interesting that while Torok and colleagues found reductions in suicidal behavior, no changes were observed in the secondary outcomes (i.e., depression and anxiety) [3], suggesting that perhaps specialized apps focused on very specific problems may be more effective than transdiagnostic approaches. With more than 10,000 mental health apps in the marketplace, finding the right app can be challenging [12]. Currently, the demand for mental health apps is driven by consumers who use online searches and social media to inform their choice of a digital mental health solution [12]. If we hope to integrate mental health apps into the routine clinical care of adolescents (i.e., to move from “code to clinic”), we need to improve healthcare professionals’ competence to select and prescribe apps safely either as stand-alone solutions to be used in place of therapy or as an adjunct to other therapies. One of the biggest obstacles to expanding adolescents’ use of apps and integrating apps into existing services are the poor adherence rates typically observed [10]. It is one thing to show that apps are effective when used under close supervision for relatively short periods in well-controlled clinical trials, but it is altogether something else to show that apps will be consistently used by adolescents in everyday settings. In this respect, the engagement rates reported by Torok and colleagues are promising, and it would be helpful if future research on the LifeBuoy app could establish why adolescents do or do not use the app consistently and how we can support them to use the technology as prescribed [10].

The significant reductions in suicidal ideation and the high engagement rates reported for LifeBuoy make this app an excellent example of how smartphones can be used as a self-guided tool to reduce suicide risk in nonclinical populations of young people with a history of suicidal behavior [3]. One paradigm for conceptualizing digital mental health interventions is to think narrowly about how technology can be used to replace humans and subsume the functions previously performed by psychotherapists. But this is not the only paradigm for conceptualizing digital interventions. Other opportunities for novel digital solutions can be identified by asking 2 alternative questions: (1) “How can technology be used to enhance or deepen relationships between adolescents’ and mental health care providers and/or facilitate connectedness?” and (2) “What can technology do that therapist cannot?”

There is little doubt about technologies’ potential to disrupt and transform adolescent mental health service delivery and to increase access to services, particularly in times of crisis such as global pandemics and conflict, when demand for services increases and conditions impede access to care. However, we still have some way to go before we realize this potential and progress will require global action. Well-designed clinical trials will move us closer, but on their own, they will not be enough. To fully realize technologies’ potential in adolescent mental health, we need to consider critical issues such as safety, appropriate regulation, prescription practices, adherence, and implementation in everyday routine care. Perhaps most importantly, we need to avoid the “AI trap” of thinking of technology narrowly as a way of replacing humans and instead think of how technology could facilitate therapeutic relationships, enhance connectedness, and perform functions that complement and reinforce interventions provided by therapists.

## Disclaimers

The views expressed here do not necessarily reflect those of the SAMRC.

## Abbreviations

app: application
COVID-19: Coronavirus Disease 2019
DBT: dialectical behavior therapy
